# Real-Time Enterovirus D68 Outbreak Detection through Hospital Surveillance of Severe Acute Respiratory Infection, Senegal, 2023

**DOI:** 10.3201/eid3008.240410

**Published:** 2024-08

**Authors:** Mamadou Malado Jallow, Marie Pedapa Mendy, Mamadou Aliou Barry, Moussa Moise Diagne, Samba Niang Sagne, Fatime Tall, Jean Baptisse Niokhor Diouf, Ndiendé Koba Ndiaye, Davy Kiori, Sara Sy, Déborah Goudiaby, Cheikh Loucoubar, Gamou Fall, Hervé Kadjo, Maël Bessaud, Ndongo Dia

**Affiliations:** Institut Pasteur de Dakar, Dakar, Senegal (M.M. Jallow, M.P. Mendy, M.A. Barry, M.M. Diagne, S.N. Sagne, N.K. Ndiaye, D. Kiori, S. Sy, D. Goudiaby, C. Loucoubar, G. Fall, N. Dia);; Hôpital des enfants Albert Royer de Fann, Dakar (F. Tall);; Hôpital Roi Baudoin de Guediawaye, Dakar (J.B.N. Diouf);; Institut Pasteur de Côte d’Ivoire, Abidjan, Côte d’Ivoire (H. Kadjo);; Institut Pasteur Paris, Paris, France (M. Bessaud)

**Keywords:** enterovirus D68, EV-D68 viruses, Dakar, Senegal, severe acute respiratory infection, SARI, surveillance, viruses

## Abstract

In December 2023, we observed through hospital-based surveillance a severe outbreak of enterovirus D68 infection in pediatric inpatients in Dakar, Senegal. Molecular characterization revealed that subclade B3, the dominant lineage in outbreaks worldwide, was responsible for the outbreak. Enhanced surveillance in inpatient settings, including among patients with neurologic illnesses, is needed.

Enterovirus D68 (EV-D68) has emerged as a major public health concern because of its association with outbreaks of severe acute respiratory illness (SARI), acute flaccid myelitis (AFM), and acute flaccid paralysis (AFP), particularly among children and persons with underlying respiratory conditions (*1*). The virus was discovered in 1962 in California, USA, in 4 children with SARI (*2*); originally named Fermon virus, it was later reclassified under enterovirus species D and serotype 68 (*3*). Before 2014, EV-D68 was reported only sporadically; a total of 699 confirmed cases in Europe, Africa, and southeast Asia were reported during 1970–2013 (*4*). However, in recent years, a notable increase in the frequency and scale of EV-D68 outbreaks has been observed, prompting heightened surveillance and public health responses. In 2014, a large outbreak of EV-D68 infection that was associated with severe respiratory illness (*5*) and, in some cases, neurologic complications such as AFP occurred in the United States and in other parts of the world (*6*). More than 2,000 cases of EV-D68 infection were reported in 20 countries during that period (*2*). After the 2014 outbreak, other waves of EV-D68 infections were observed in 2016, 2018, and 2022; outbreaks were reported in several parts of the world, including the countries Sweden (*7*), Japan (*8*), and Finland (*9*).

In Senegal, few EV-D68 infection cases were detected in 2014 (*10*). In 2016, an outbreak of novel subclade B3 infections in outpatients with influenza-like illness and AFP (*11*) were reported. Since 2016, the virus has been detected sporadically through community surveillance of respiratory infections until December 2023, when a notable upsurge of EV-D68 infections in pediatric inpatients occurred. We report on an outbreak of severe EV-D68 infections in pediatric patients in Dakar, Senegal, and describe the clinical characteristics of EV-D68 cases identified in this outbreak.

## The Study

**Figure 1:**
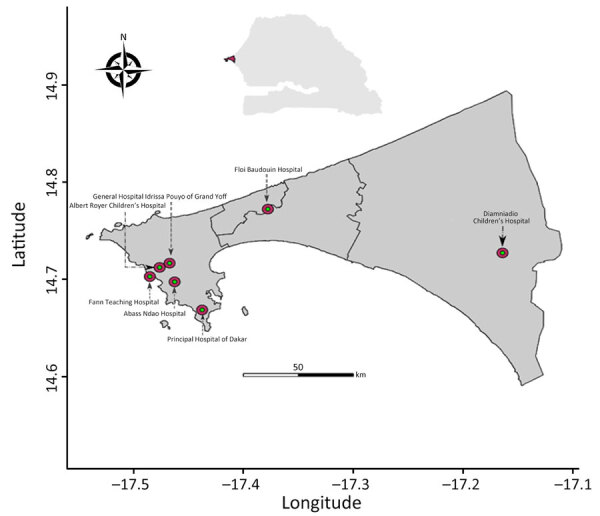
Referral hospitals contributing to the hospital-based surveillance of severe acute respiratory infection (red circles), Dakar, Senegal, 2023. Inset map show study area in Senegal.

In 2015, in collaboration with Senegal’s Ministry of Health, the Institut Pasteur of Dakar initiated in hospital-based surveillance of SARI in referral hospitals in the capital of Dakar through its National Influenza Centre (NIC) and its Unit of Epidemiology Clinical Research and Data Sciences (Figure 1) to enable the Ministry of Health to quickly detect and alert any of abnormal health event. As part of this routine surveillance, which falls within the scope of the Sentinel Syndromic Surveillance in Senegal’s network activities, we collected swab samples (nasopharyngeal, oropharyngeal, or both) from hospitalized patients with SARI and promptly transported the specimens at a controlled temperature (4°C–8°C) to NIC to screen for respiratory pathogens, as detailed by Jallow et al. (*12*). We then typed all enterovirus-positive samples by using 1-step real-time reverse transcription PCR (rRT-PCR) by using primers and a probe specific to EV-D68, as previously described (*10*). We genetically characterized all EV-D68 isolates by using whole-genome sequencing on an Illumina sequencing platform (https://www.illumina.com) with the Twist Respiratory Virus Research Panel (Twist Biosciences, https://www.twistbioscience.com), as previously described (*12*). This study was conducted as part of SARI hospital-based surveillance with approval from the National Ethical Committee of Senegal’s Ministry of Health.

**Figure 2:**
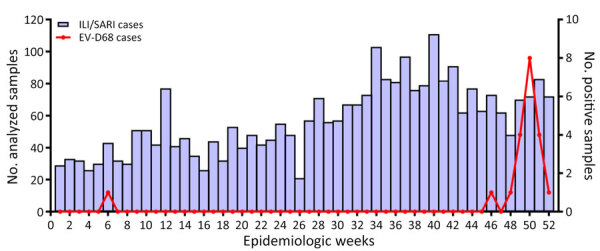
Weekly distribution of EV-D68 in patients with acute respiratory infection, Dakar Senegal, 2023. Bars represent the number of samples tested for each epidemiologic week. Line indicates number samples positive for EV-D68. Scales for the y-axes differ substantially to underscore patterns but do not permit direct comparisons. EV-D68, enterovirus D68; ILI, influenza-like illness; SARI, severe acute respiratory illness.

In December 2023, NIC noticed an increase in the positivity rate of enterovirus (0% in January, 1.1% from February to November, and 6.9% in December) in SARI samples, even though the number of specimens tested monthly remained relatively unchanged. The typing of enterovirus-positive samples identified EV-D68 as the predominant virus implicated in this upsurge of cases. Of the 2,986 nasopharyngeal samples from patients with influenza-like illness and SARI collected at the different sentinel sites during January–December 2023, a total of 45 (1.5%) tested positive for enterovirus (Appendix). We detected EV-D68 in 20 (44.4%) of the enterovirus-positive specimens, and most cases (8 [40%]) were recorded during epidemiologic week 50 (Figure 2). The proportion of specimens in which EV-D68 was detected increased from 0.04% during February–September (1/2,338) to 0.7% in November (2/295) and 5.8% in December (17/291). The 20 patients in whom EV-D68 was detected were all children <6 years of age (median age 2 years); 65% (13/20) of cases were in girls and 35% (17/20) in boys. Almost all children with confirmed EV-D68 infection (18/20 [90%]) were inpatients with SARI who had been admitted to 2 referral hospitals, Roi Baudouin hospital (11 [55%]) in a suburb of Dakar and Albert Royer Children’s Hospital (7 [35%]) in Dakar. The primary reason for hospitalization was bronchiolitis for 30% (6/20), acute bronchitis for 10% (2/20), pneumonia for 30% (6/20), and asthma exacerbation for 20% (4/20). One patient was found to have an underlying medical condition (prematurity). Nearly half (9 [45%]) of children with confirmed EV-D68 infection needed supplemental oxygen. The common clinical characteristics at the time of admission were cough (15 [75%]), breathing difficulties (15 [75%]), fever (9 [45%]), wheezing (5 [25%]), tachypnea (4 [20%]), rhinitis (3 [15%]), and apnea (2 [10%]). Although EV-D68 can cause AFM and other neurologic complications, children infected with EV-D68 during this outbreak had no neurologic symptoms, unlike those described in reports from Europe (*13*) and the United States (Colorado) in 2014 (*14*).

Eight children were co-infected with rhinovirus (6 children), bocavirus (1 child), or human metapneumovirus (1 child). We also encountered mixed infections with *Haemophilus infuenzae* (2 children) and *Streptococcus pneumoniae* (2 children).

**Figure 3:**
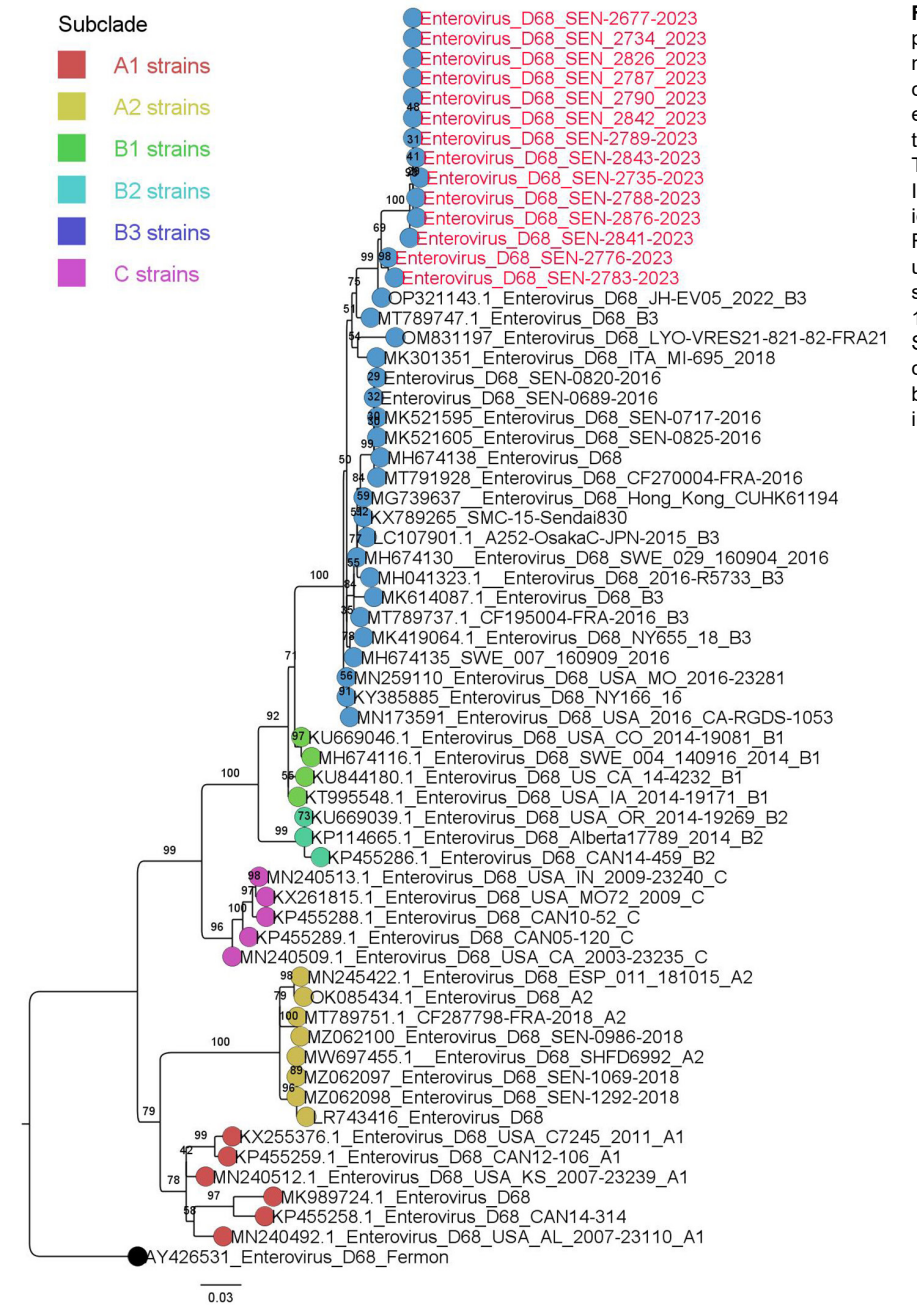
Maximum-likelihood phylogenetic tree based on the nucleotide sequences of major capsid protein gene region of enterovirus D68 from Senegal (red text) and reference sequences. Tree was constructed by using IQ-TREE2 2.0.6 (http://www.iqtree.org) and visualized by using Figtree 1.4.4 (http://tree.bio.ed.ac.uk/software/figtree). Statistical significance was tested by using 1,000 bootstrapping replicates. Software was used to define the correct model used. Tree is rooted by the Fermon strain. Scale bar indicates substitutions per site.

For the molecular characterization, we successfully obtained 14 complete EV-D68 genomes and deposited them into GenBank (accession nos. PP838726–39). We initially used the enterovirus online genotyping tool (https://www.rivm.nl/mpf/typingtool/enterovirus) for genotype predictions of EV-D68 strains. The genotyping tool classified all EV-D68 sequences into the sub-genogroup B3. We undertook phylogenetic analysis to further confirm this assignment. The maximum-likelihood phylogenetic tree based on major capsid protein gene region sequences clearly shows that all EV-D68 strains from this outbreak belonged to the B3 lineage, which has been the main subclade of EV-D68 circulating in Senegal since 2016 (Figure 3). However, this B3 lineage circulated at a very low level in 2022, when the A2 subclade emerged and was the dominant strain. By using BLAST (https://blast.ncbi.nlm.nih.gov), we found that the B3 strains from our study were closely related to B3 strains of EV-D68 that circulated in the United States (Maryland) in 2022 (*15*), showing nucleotide similarity of 97.8%–99.37%.

One limitation of this study is that almost all cases of EV-D68 were detected on the basis of samples collected from only 2 referral hospitals in Dakar, which might not reflect the actual incidence of EV-D68 infection for this outbreak. Therefore, active surveillance in inpatient settings across more areas would probably give a more accurate picture of EV-D68 infection in Senegal. Despite this limitation, our study identified an outbreak of EV-D68 in Senegal in real-time, whereas all previous cases were identified in retrospective studies.

## Conclusions

We observed a sudden onset of an EV-D68 outbreak in Dakar that was exceptionally intense and lasted from epidemiologic weeks 48 through 52 of 2023. The outbreak was caused by the B3 lineage, which has been circulating in Senegal since 2016, and all infected patients were children <6 years of age, most of whom required hospitalization. Given the ability of EV-D68 to cause AFM and AFP, this upsurge of EV-D68 infections in pediatric inpatients underscores the need for enhanced surveillance in inpatient settings across more areas, including collecting respiratory specimens from patients with neurologic illnesses. 

Appendix Additional information about real-time enterovirus D68 outbreak detection through hospital surveillance of severe acute respiratory infection, Senegal, 2023.
