# Supplementary material for: Real-Time Enterovirus D68 Outbreak Detection through Hospital Surveillance of Severe Acute Respiratory Infection, Senegal, 2023
Source: Emerg Infect Dis. 2024 Aug;30(8):1687–91. doi: 10.3201/eid3008.240410 (PMC11286061; doi:10.3201/eid3008.240410)
Supplement: Appendix — Additional information about real-time enterovirus D68 outbreak detection through hospital surveillance of severe acute respiratory infection, Senegal, 2023. [file 24-0410-Techapp-s1.pdf]

Article DOI: https://doi.org/10.3201/eid3008.240410

EID cannot ensure accessibility for supplementary materials supplied by authors. Readers who have difficulty accessing supplementary content should contact the authors for assistance.

Real-Time Enterovirus D68 Outbreak Detection through Hospital Surveillance of Severe Acute Respiratory Infection, Senegal, 2023

Appendix

Appendix Table 1. Viral pathogens detected as part of the sentinel surveillance of SARI in Senegal in 2023

	SARI	ILI	Total
Viral ARI Pathogen, no. (%)	N = 949	N = 2,037	N = 2,986
Enterovirus	43 (4.5)	2 (0.1)	45 (1.5)
Influenza H3N1	1 (0.1)	36 (1.8)	37 (1.2)
Influenza H1N1pdm09	27 (2.8)	454 (22.3)	481 (16.1)
Influenza B	1 (0.1)	2 (0.1)	3 (0.1)
Adenovirus	40 (4.2)	135 (6.6)	175 (5.9)
Bocavirus	28 (2.9)	0 (0.0)	28 (0.9)
HCoV-229E	6 (0.6)	0 (0.0)	6 (0.2)
HCoV-NL63	11 (1.16)	0 (0.0)	11 (0.4)
HCoV-OC43	12 (1.3)	0 (0.0)	12 (0.4)
HMPV	31 (3.3)	76 (3.7)	107 (3.6)
PIV	45 (4.7)	100 (4.9)	145 (4.8)
Rhinovirus	175 (18.4)	377 (18.5)	552 (18.5)
SARS-CoV-2	30 (3.2)	128 (6.3)	185 (6.2)
RSV	125 (13.2)	212 (10.4)	337 (11.3)

ARI = Acute Respiratory Infection; SARI = Severe Acute Respiratory Infection; ILI = Influenza-like Illness

Appendix Table 2. Bacterial pathogens detected among patients with severe acute respiratory infections (SARI) in Senegal in 2023

Bacterial Pathogen	Positive (N = 429), no. (%)
Bordetella parapertussis	2 (0.5)
Bordetella pertussis	16 (3.7)
Chlamydophila pneumonia	2 (0.5)
Haemophilus Influenza	212 (49.4)
Mycoplasma pneumoniae	13 (3.0)
Streptococcus pneumoniae	183 (42.6)
Morax Ella pneumoniae	1 (0.2)

Appendix Table 3. Information on patients with confirmed EV-D68 infection in Senegal, 2023

						Clinical symptoms						
ID No	Sex	Age	Onset date	Admission date	Clinical diagnosis	Cough	Dyspnea	Fever	Wheezing	Tachypnea	Rhinitis	Apnea
230212	F	2 m	09/02/2023	12/02/2023	Pneumonia	+	+	+	–	+	–	–
232594	M	2 y	14/11/2023	15/11/2023	Pneumonia	+	+	+	–	–	–	–
232677	F	11 m	26/11/2023	27/11/2023	Bronchiolitis	+	+	–	–	–	+	–
232734	M	5 m	02/12/2023	03/12/2023	Bronchiolitis	+	+	–	–	–	–	–
232735	F	2 y	06/12/2023	07/12/2023	asthma exacerbation	+	+	+	+	–	–	–
232776	F	1 y	08/12/2023	09/12/2023	Bronchiolitis	–	+	–	–	–	–	–
232783	F	1 y	08/12/2023	11/12/2023	acute bronchitis	–	+	+	–	+	–	–
232787	M	3 y	08/12/2023	08/12/2023	Pneumonia + asthma	+	+	–	+	–	–	–
232788	F	1 y	12/12/2023	12/12/2023	Pneumonia	+	+	+	–	–	–	–
232789	F	6 y	11/12/2023	12/12/2023	asthma exacerbation	–	+	+	+	–	–	+
232790	F	4 y	07/12/2023	08/12/2023	asthma exacerbation	+	+	–	+	–	–	+
232826	F	2 y	11/12/2023	12/12/2023	ILI	+	–	+	–	–	–	–
232841	F	6 y	16/12/2023	17/12/2023	Bronchiolitis	+	+	–	–	–	–	–
232842	M	2 y	13/12/2023	17/12/2023	Bronchiolitis	+	+	–	+	–	–	–
232843	M	2 y	17/12/2023	19/12/2023	Bronchiolitis	+	+	–	–	–	–	–
232874	F	6 y	13/12/2023	14/12/2023	Severe acute asthma	+	–	–	–	+	–	–
232876	M	1 y	15/12/2023	15/12/2023	Pneumonia	+	–	+	–	+	–	–
232897	F	3 y	20/12/2023	20/12/2023	Pneumonia	+	+	–	–	–	–	–
232926	M	2 y	12/12/2023	15/12/2023	ILI	–	–	+	–	–	+	–
232939	F	1 y	22/12/2023	25/12/2023	acute bronchitis	–	–	–	–	–	+	–

ID No. Identification number; y, years; m, months; +, positive; −, negative
